# Cdc7 is a potent anti-cancer target in pancreatic cancer due to abrogation of the DNA origin activation checkpoint

**DOI:** 10.18632/oncotarget.7611

**Published:** 2016-02-23

**Authors:** Matthew T. Huggett, Slavica Tudzarova, Ian Proctor, Marco Loddo, Margaret G. Keane, Kai Stoeber, Gareth H. Williams, Stephen P. Pereira

**Affiliations:** ^1^ UCL Institute for Liver and Digestive Health and UCL Cancer Institute, University College London, London, UK; ^2^ The Research Department of Pathology, UCL Cancer Institute, University College London, London, UK; ^3^ Oncologica Ltd, The Science Village, Chesterford Research Park, Cambridge, UK

**Keywords:** pancreatic cancer, cell cycle, DNA replication, Cdc7

## Abstract

**Purpose:**

Cdc7 is a serine/threonine kinase which is responsible for the ‘firing’ of replication origins leading to initiation of DNA replication. Inhibition or depletion of Cdc7 in normal cells triggers a DNA origin activation checkpoint causing a reversible G1 arrest. Here we investigate Cdc7 as a novel therapeutic target in pancreatic cancer.

**Experimental design:**

Cdc7 target validation was performed by immunoexpression profiling in a cohort of 73 patients with pancreatic adenocarcinoma including 24 controls. Secondly Cdc7 kinase was targeted in Capan-1 and PANC-1 pancreatic cancer cell line models using either an siRNA against Cdc7 or alternatively a small molecule inhibitor (SMI) of Cdc7 (PHA-767491).

**Results:**

Cdc7 was significantly overexpressed in pancreatic adenocarcinoma compared to benign pancreatic tissue (median LI 34.3% vs. 1.3%; P<0.0001). Cdc7 knockdown using siRNA in Capan-1 and PANC-1 cells resulted in marked apoptotic cell death when compared with control cells. A prominent sub-G1 peak was seen on flow cytometry (sub-G1 51% vs. 3% and 45% vs. 0.7% in Capan-1 and PANC-1 cells, respectively). Annexin V labelling confirmed apoptosis in 64% vs. 11% and 75% vs. 8%, respectively. Western blotting showed cleavage of PARP-1 and caspase-3 and presence of γH2A.X. TUNEL assay showed strong staining in treated cells. These results were mirrored following Cdc7 kinase inhibition with PHA-767491.

**Conclusions:**

Our findings show that Cdc7 is a potent anti-cancer target in pancreatic adenocarcinoma and that Cdc7 immunoexpression levels might be used as a companion diagnostic to predict response to therapeutic siRNAs or SMIs directed against this kinase.

## INTRODUCTION

Adenocarcinoma of the pancreas is one of the top 10 leading causes of cancer deaths [1, 2]. Surgical resection provides potential for cure but is possible in only a minority of subjects. Even after resection, the median survival is limited to 10–20 months and only 10-35% of resected patients survive five years or more [3–6]. Palliative chemotherapy is associated with limited improvements in quality of life and survival when compared with best supportive care [7, 8], but despite improvements in imaging, surgical techniques and chemotherapy, overall survival has not improved appreciably in the last few decades [1, 2].

An alternative approach for the treatment of pancreatic cancer is the targeting of the DNA replication initiation machinery which acts as a convergence point for upstream oncogenic signaling pathways [9]. The initiation of DNA synthesis can be regarded as a final and critical step in growth regulatory control and therefore a potentially potent anti-cancer target [10]. Cdc7 kinase is a core component of this machinery and is therefore an attractive target for therapeutic intervention strategies. During late mitosis and early G_1_ phase, the replication licensing factors (RLF) ORC, Cdc6, Cdt1, and Mcm2-7 assemble into prereplicative complexes (pre-RCs), which render replication origins “licensed” for DNA synthesis during S phase [11, 12]. The six Mcm2-7 proteins function as a replicative helix, unwinding the template DNA, with Cdc6 and Cdt1 acting as clamp loaders for this ring-shaped heterohexameric complex [13–15]. At the transition from G1 to S phase, licensed replication origins are “fired” by the concerted action of CDKs and Dbf4-dependent kinase. Cdc7 phosphorylates the Mcm 2,4 and 6 subunits, thereby inducing a conformational change that stimulates MCM helicase activity. The formation of an active helicase leads to recruitment of additional factors, including Cdc45 and the four subunit GINS complex [16–18]. Once activated the MCM helicase unwinds double-stranded DNA at replication origins to generate a single-stranded DNA template required to recruit the DNA synthesis machinery including RPA, PCNA and DNA polymerase α-primase [12]. During S phase, Cdc7 kinase and cyclin-dependent kinases induce a conformational change in the prereplicative complex, resulting in recruitment of additional initiator proteins that collectively promote DNA unwinding, recruitment of DNA polymerases and the establishment of bi-directional replication forks.

**Figure 1:**
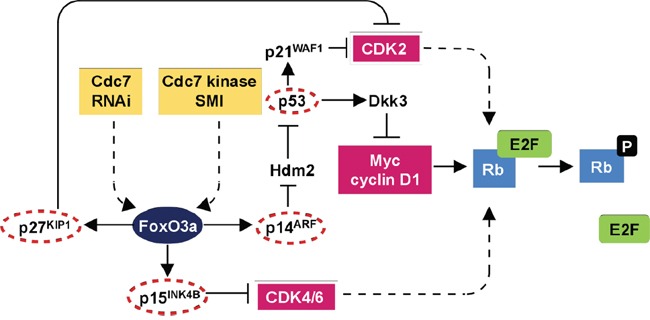
DNA origin activation checkpoint The checkpoint response is dependent on three effector axes coordinated through the transcription factor FoxO3a. In arrested cells, FoxO3a activates the ARF-|Hdm2-|p53→p21 pathway and mediates p15(INK4B) upregulation. p53 in turn activates expression of the Wnt/β-catenin signalling antagonist Dkk3, leading to Myc and cyclin D1 downregulation. The resulting loss of CDK activity inactivates the Rb-E2F pathway resulting in G1 arrest. The lack of redundancy between the checkpoint axes and reliance on several tumour suppressor proteins commonly inactivated in human tumours provides a mechanistic basis for the cancer-cell-specific killing observed following targeting of Cdc7. Notably multiple core components of this checkpoint pathway are mutated in pancreatic cancer (circled in red) thus sensitizing this tumour type to Cdc7 directed agents (adapted from Tudzarova et al, EMBO J. 2010 Oct 6;29(19):3381-94, Figure 6).

The interest in Cdc7 as a therapeutic target comes from the observation that depleting Cdc7 levels using siRNA, or alternatively inhibiting Cdc7 kinase activity in cancer cells, results in only limited numbers of replication forks being established during S phase, culminating in fork stalling and/or collapse followed by apoptotic cell death [19–22]. Intriguingly, the specificity of this approach relates to the fact that normal somatic cells avoid entering a lethal S phase under Cdc7 rate limiting conditions by engaging a DNA origin activation checkpoint response that reversibly arrests cells in G1 phase until Cdc7 levels are restored following removal of the Cdc7 targeting agent [19, 23]. Our studies and those of other groups have previously shown that this checkpoint is p53 dependent [19–21, 23]. More recently we have determined that this checkpoint is also dependent on a number of additional tumour suppressor genes commonly inactivated in solid tumours [23]. We have shown that this DNA checkpoint response is mediated via three signalling axes coordinated through the transcription factor FoxO3a (Figure 1). In arrested cells, FoxO3a activates the ARF-|Hdm2-|p53-p21 pathway and mediates p15INK4B upregulation; p53 in turn activates expression of the Wnt/b-catenin signalling antagonist Dkk3, leading to Myc and cyclin D1 downregulation. The resulting loss of CDK activity inactivates the Rb-E2F pathway and overrides the G1-S transcriptional programme. Notably we observed no redundancy in these checkpoint effector axes, meaning that any tumours harbouring inactivating mutations in any of the tumour suppressor genes namely p53, p21, Dkk3, ARF, Hdm2, FoxO3a, p15, p27 and Rb are susceptible to Cdc7 targeting agents. Taken together these findings indicate that targeting Cdc7 constitutes a synthetic lethality driven approach in tumour backgrounds harbouring commonly mutated tumour suppressor genes.

Interestingly, inactivating mutations in p53 not only disrupt the DNA origin activation checkpoint making tumours susceptible to targeting of Cdc7, but also result in increased expression of the Cdc7 anti-cancer target thus potentially increasing therapeutic efficacy. In a study of 62 human cancer cell lines, Cdc7 was found to be increased in ~50% of these lines relative to β-actin with levels in normal primary cell lines either very low or absent [24]. Notably, a strong association between high Cdc7 expression levels and mutated TP53 was observed in which 90% of mutant p53 cancer cell lines overexpressed Cdc7.

The cancer specific killing following Cdc7 targeting has generated great interest in the development of therapeutic Cdc7 small molecule inhibitors. For example, PHA-767491 (Nerviano Medical Sciences, Nerviano, Italy), a dual Cdc7/Cdk9 inhibitor, has been shown to have activity by inducing apoptosis in a wide variety of cancer cell lines. Furthermore, in human HL60 leukemia human tumours implanted subcutaneously into nude mouse models, there was inhibition of tumour growth, and even regression of tumours in some animals, when treated with PHA-767491 [22]. In another study, chronic lymphocytic leukaemia (CLL) cells were taken from the peripheral blood of patients with CLL and treated with PHA-767491. In all samples taken from 27 different patients with both favourable and unfavourable prognostic markers, PHA-767491 induced apoptosis of the CLL cells [25].

The recent advances in our understanding of the DNA replication initiation machinery and its clinical translational exploitation in terms of anti-cancer therapy raise the important question as to whether targeting Cdc7 might be of clinical benefit in pancreatic cancer for which therapeutic options are limited. Importantly, 70% of pancreatic adenocarcinoma tumours have been shown to have inactivating mutations of p53, one of the highest frequencies amongst all cancers for this tumour suppressor [26, 27]. Moreover, pancreatic cancers commonly harbor mutations in additional tumour suppressor genes encoding for p14^ARF^ and p16^INK4A^, p15^INK4B^ and p27^Kip1^; key constituent proteins encompassing all three effector arms of the DNA origin activation checkpoint [28–33]. It might therefore be postulated that these tumours will be highly sensitive to Cdc7 targeted therapies and secondly that the Cdc7 target protein will be expressed at high levels in this tumour type. In this study we therefore set out to assess expression of the target protein Cdc7 in pancreatic cancer (target validation) and to test therapeutic anti-cancer potential by targeting Cdc7 kinase in pancreatic cancer cell line models using both siRNA and the PHA-767491 small molecule inhibitor (SMI) treatment strategies.

## RESULTS

### Immunohistochemistry

**Figure 2:**
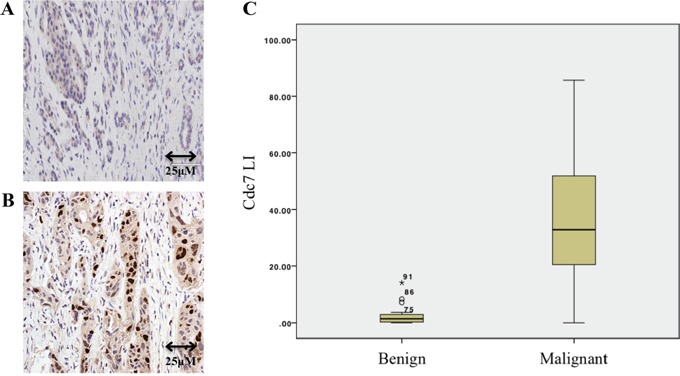
Immunohistochemistry of benign A. and malignant B. pancreatic tissue showing Cdc7 staining **C.** Box and whisker plot showing that the Cdc7 labelling index (LI) in patients with pancreatic cancer was significantly higher than in those who had resections for benign disease (P<0.0001).

Seventy three patients with pancreatic ductal (n=62) and ampullary (n=11) cancer were included in the study cohort with a control set of 24 patients with benign pancreatic disease. Immunohistochemical analysis showed markedly higher expression levels of Cdc7 protein in pancreatic ductal and ampullary cancers when compared with benign pancreatic disease, in which Cdc7 was detected in only very small numbers of cells (median LI 34.3%, IQR 28.6 to 63.4% vs. median LI 1.3%, IQR 0.3 to 2.9%; P<0.0001) (Figure 2).

It was found that when the Cdc7 levels were assessed in biopsy samples and in resection samples for pancreatic cancer, higher expression was seen in the resection samples. To explain this. it was noted that in biopsy specimens there was often only a small amount of tumour tissue included, surrounded by stroma and inflammatory cells. As the protocol was to identify the regions with the highest expression and to photograph these (‘hot-spot’ technique), there may have been significant sampling error in the biopsy only specimens. Analysis was thus carried out following adjustment for several potential confounding variables (age, sex, tumour differentiation, ampullary vs. pancreatic ductal origin, resection status presence of metastatic disease). The association between Cdc7 expression and patient survival was examined for a 10-point increase in labeling. No association was seen between the Cdc7 labeling index and patient survival in the corrected analysis (Supplementary table).

Cdc7 expression was also compared to 3 other markers for cell cycle progression-Mcm2, geminin and phosphohistone H3. Spearman's Rank correlation coefficient was used to assess the association between the markers with both biopsies and pancreatic resections included. All markers were strongly associated with one another, with P<0.0001 for each correlation (Supplementary Figure 1). Importantly, Cdc7 was found to be strongly positively correlated with the other markers, confirming that actively cycling cells have high levels of Cdc7 and that Cdc7 is a potential therapeutic target in pancreatic ductal and ampullary adenocarcinoma.

### *CDC7* knockdown in PANC-1 and Capan-1 pancreatic adenocarcinoma cell lines

**Figure 3:**
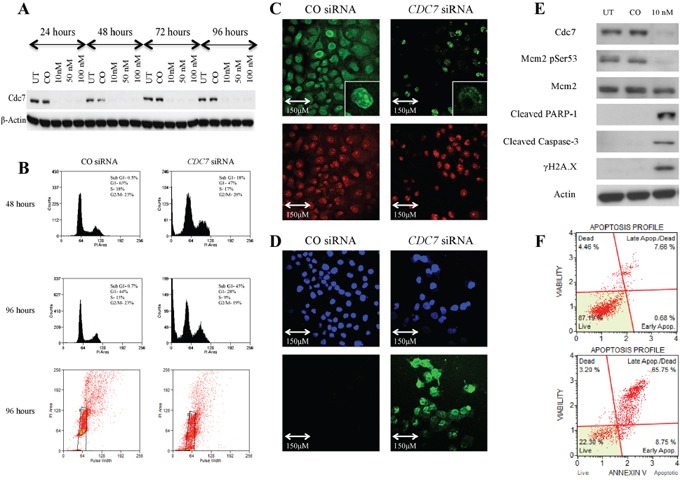
Knockdown of *CDC7* mRNA in PANC-1 pancreatic adenocarcinoma cells following transfection with custom siRNA **A.** Western blot showing Cdc7 protein expression levels following transfection with different concentrations of *CDC7* siRNA (10, 50 and 100 nM) along with untreated (UT) and non-coding siRNA (CO). There was evidence of reduced protein expression at each concentration and at each time point. β-Actin loading control is shown below. **B.** Flow cytometry showing that at 48 hours, there was no enrichment of PANC-1 cells in the G1 population following treatment with 10 nM *CDC7* siRNA, and cells started to accumulate in a sub G1 peak. At 96 hours this effect was more pronounced with evident cell death, as represented by the sub G1 peak of 45% and plot of distribution including examples of gating. **C.** BrdU staining (green) in cells treated with 10 nM *CDC7* siRNA and CO siRNA. A much smaller proportion of the *CDC7* siRNA cells stained positive, indicating reduced synthesis of new DNA. PI staining (red) is shown as a control. **D.** There was avid TUNEL staining (green) of 10 nM *CDC7* siRNA treated cells indicating apoptosis. DAPI staining (blue) is shown as a control. **E.** Western blot showing protein levels at 96 hours following Cdc7 depletion with 10 nM siRNA in the PANC-1 pancreatic adenocarcinoma cell line. There was reduced expression of Cdc7 protein and also loss of Cdc7 target phosphorylation of Mcm2 at Ser53. There was evidence of activation of the classical apoptotic pathway with cleavage of PARP-1 and Caspase-3. Phosphorylated γH2A.X was seen after Cdc7 depletion suggesting double strand DNA breaks. **F.** Annexin V staining confirmed apoptosis (early and late) in 8% of CO siRNA treated cells (upper graph), compared with 75% of the 10 nM *CDC7* siRNA treated cells (lower graph).

There was efficient Cdc7 mRNA knockdown in each cell line, with a mean reduction of 90% and 95% after 48 hours, in PANC-1 and Capan-1 cells respectively, when compared to transfection with the control siRNA (Supplementary Figure 2). Cdc7 protein levels were also reduced to an undetectable level as assessed by western blot, as was Mcm2 phosphorylated on serine 53, which serves as a biomarker for Cdc7 functionality (Figures 3A-3E and Supplementary Figure 3A-3E). Next the effect of Cdc7 depletion in both lines was assessed by FACS flow cytometry. In Figure 3B and Supplementary Figure 3B, DNA histograms of the *CDC7* knockdown cells show the appearance of a sub-G1 peak of cells which had less than 2C DNA content. This effect became more pronounced over time and, after 96 hours, 45% of PANC-1 and 51% of Capan-1 cells had accumulated in the sub-G1 peak, compared with less than 3% of control cells.

When protein levels were analysed after 96 hours, there was reduced Cdc7-target phosphorylation of Mcm2 on Serine 53, consistent with loss of kinase activity. Increased expression of the cleavage products of both PARP-1 (89 KDa) and Caspase-3 (17 KDa) was coupled to loss of Cdc7 kinase activity indicating induction of the classical apoptotic pathway (Figure 3E and Supplementary Figure 3E). The triggering of apoptotoic cell death was confirmed by the observation that H2A.X serine 139 phosphorylation (known as γH2A.X), was found to be increased in the Cdc7 knockdown cells, compared with the control siRNA. H2A.X is phosphorylated at this site in response to DNA double strand breaks which occur during genotoxic stress [41, 42].

**Figure 4:**
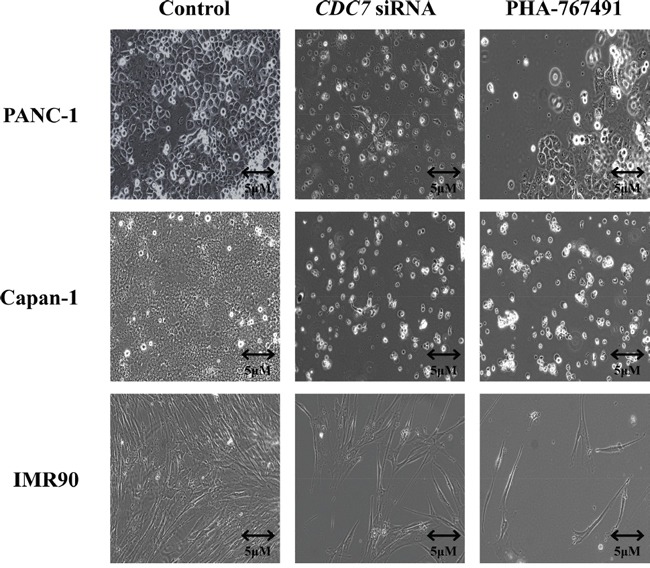
Phase contrast light microscopy of PANC-1, Capan-1 or IMR90 cells treated with either siRNA or PHA-767491, compared with controls Images of cells obtained at 200X magnification using a Zeiss AxioObserver A1 microscope. 96 hours following treatment with either CO siRNA, DMSO control or untreated typically growing PANC-1, Capan-1 and IMR90 cells were seen (representative images shown as a control). Following treatment with *CDC7* siRNA or PHA-767491 the cancer cells became detached from the culture vessel and were seen freely floating within the media. In contrast IMR90 cells treated with *CDC7* siRNA or PHA-767491 did not change their morphology but instead appeared to arrest growth with no increase in population.

When viewed with a phase contrast microscope, cells treated with *CDC7* siRNA appeared to lose their normal cell-cell interactions, exhibited widespread cell death, and became detached from the culture flasks (Figure 4). In Figure 3C and Supplementary Figure 3C both *CDC7* siRNA and CO siRNA cells stained for BrdU, but a much smaller proportion of the *CDC7* siRNA cells stained positive, indicating reduced synthesis of new DNA and consistent with DNA fork stalling in the presence of rate limiting levels of Cdc7 kinase activity.

Two additional methods were used to specifically demonstrate apoptosis in the cells. Terminal deoxynucleotidyl transferase labelling (TUNEL) recognises nicks in the DNA molecule to which the TdT enzyme incorporates fluorescently labelled nucleotides. TUNEL staining occurs in cells in the later stages of apoptosis. In Figure 3D and Supplementary Figure 3D strong staining can be seen in the *CDC7* siRNA treated cells, with all cells having at least some staining (DAPI used as nuclear stain). In contrast no staining in the CO siRNA treated cells was observed.

Figure 3F and Supplementary Figure 3F show Annexin V labelled cells quantified using flow cytometry. Annexin V recognises the phospholipid phosphatidylserine which is translocated onto the outer cell membrane as an early event following the triggering of apoptosis in cells. 75% and 64% (PANC-1 and Capan-1 respectively) of the Cdc7 depleted cells were found to stain positively for Annexin V, compared to approximately 10% of cells treated with control siRNA.

Cells were also treated with an alternative *CDC7* siRNA to control for a non-target effect shown in the original experiments (data not shown). *CDC7* knockdown with the alternative siRNA also showed efficient reduction in mRNA levels and protein analysis by western blot showed the same phenotypic pattern as with the original siRNA, with appearance of cleaved PARP-1, cleaved Caspase-3 and γH2A.X; confirming induction of the classical apoptotic cascade and DNA double-strand breaks. FACS analysis demonstrated that Cdc7 depletion with the alternative siRNA resulted in development of a strong sub-G1 peak, compared to a negligible proportion of cells treated with a non-coding control. These results show that the effect of Cdc7 depletion using 2 different *CDC7* siRNAs have the same functional effect on these cell lines.

### *CDC7* knockdown in IMR-90 cell line

**Figure 5:**
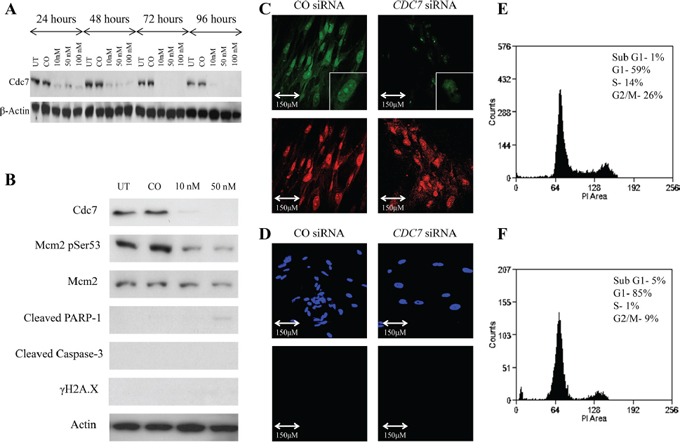
Knockdown of *CDC7* mRNA in IMR90 fibroblast cells following transfection with custom siRNA **A.** Western blot showing Cdc7 protein expression levels following transfection with different concentrations of *CDC7* siRNA (10, 50 and 100 nM) along with untreated (UT) and non-coding siRNA (CO). There was evidence of reduced protein expression at each concentration and at each time point. β-Actin loading control is shown below. **B.** Western blot showing protein levels at 96 hours following Cdc7 depletion in the IMR90 fibroblast cell line. There was reduced expression of Cdc7 protein and also loss of Cdc7 target phosphorylation of Mcm2 at Ser53. In contrast to the changes seen in the cancer cells, there was no evidence of activation of the classical apoptotic pathway with no staining for cleaved PARP-1, Caspase-3 or γH2A.X. **C.** BrdU assay demonstrated reduced staining of the *CDC7* siRNA treated cells (top images, green) when compared with propidium iodide (bottom images, red), whereas the control siRNA cells stained for both BrdU and PI. These findings indicate reduced synthesis of new DNA when the cells were treated with *CDC7* siRNA. **D.** TUNEL assay showed no evidence of TUNEL labelling in CO siRNA or *CDC7* siRNA treated cells (DAPI top, blue; TUNEL bottom, green). This assay was performed at the same time and under the same conditions as *CDC7* siRNA treatments of PANC-1 and CAPAN-1 cells, which served as the positive control. On FACS analysis, treatment of IMR-90 fibroblasts with *CDC7* siRNA **F.** induced an accumulation of cells in the G1 population, compared to CO siRNA treated cells **E.** (85% vs. 59%). There was a minimal sub-G1 peak (5% vs. 1%) seen. These findings suggest that Cdc7 depletion causing a G1 arrest in IMR90 cells.

To confirm previous observations that untransformed cells would exhibit a G1 arrest in response to *CDC7* knockdown, the IMR-90 human diploid fibroblast line derived from fetal lung tissue was used. There was efficient knockdown of *CDC7* mRNA when using 10 nM *CDC7* siRNA in IMR-90 fibroblasts. mRNA levels were reduced by a mean of 83% in these experiments, as demonstrated by qRT-PCR. Western blotting confirmed loss of Cdc7 protein expression at each concentration used, with undetectable protein by 72 hours (Figure 5A). In parallel with these observations, Mcm2 was found to be hypophosphorylated on serine 53 when compared to control cells, confirming the reduced functional effect of Cdc7 kinase. There was minimal detection of cleaved PARP-1 at 89 KDa (none detectable at 10 nM) and no expression of cleaved Caspase-3 or of γH2A.X (Figure 5B). On phase contrast microscopy, cells showed significantly reduced confluency when compared to the control cells (Figure 4). BrdU staining was almost absent in *CDC7* siRNA, indicating a powerful G1 cell cycle arrest and failure of progression into S phase (Figure 5C). In keeping with these observations there was a complete absence of staining by the TUNEL assay indicating that these cells remain viable in this arrested state and confirming previous studies with untransformed cells lines (Figure 5D).

FACS flow cytometry showed that, in the *CDC7* siRNA treated cells, there was a functional cell cycle arrest with 85% of cells accumulating in G1 phase, and with only 1% and 9% progressing through to S and G2/M phases respectively (Figure 5E and 5F). This was in contrast with CO siRNA treated cells in which 59% of the population were in G1 phase, with 14% in S phase and 26% in G2/M phases. Only a small fraction of cells was observed with a sub-G1 peak (5% and 1%, respectively).

### Treatment of pancreatic adenocarcinoma cell lines and IMR-90 fibroblasts with the Cdc7/Cdk9 kinase inhibitor PHA-767491

**Figure 6:**
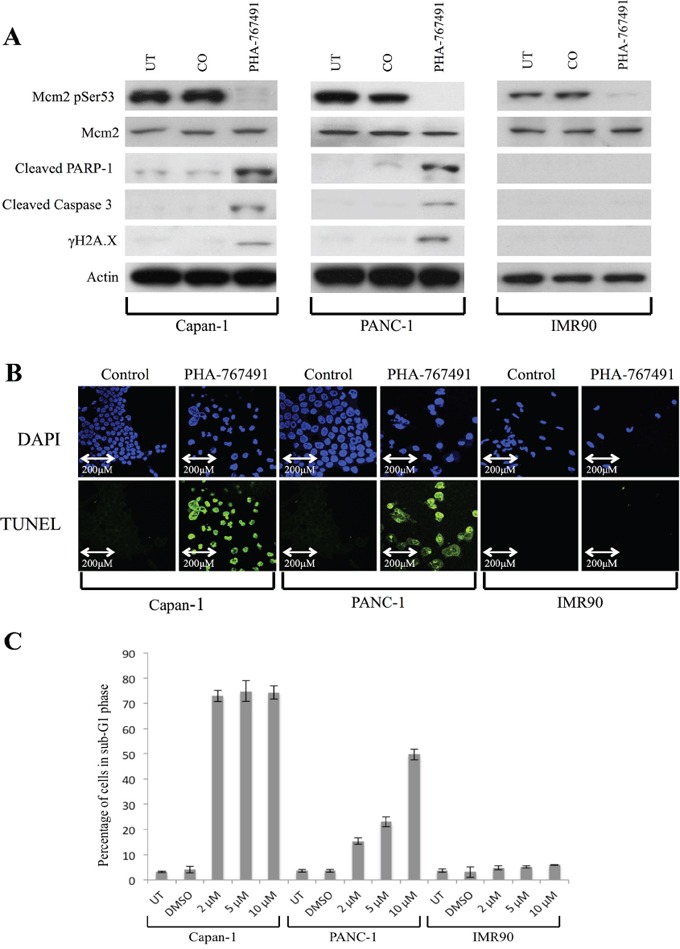
Effect of treatment with PHA-767491 on Capan-1, PANC-1 and IMR90 cell lines **A.** Western blot showing protein levels at 96 hours following Cdc7/Cdk9 inhibition in each cell line. There was loss of Cdc7 target phosphorylation of Mcm2 at Ser53 in all lines. There was evidence of activation of the classical apoptotic pathway with cleavage of PARP-1 and Caspase-3 in the pancreatic cancer lines, but not in IMR90. Furthermore, in the cancer lines, phosphorylated γH2A.X was seen after treatment with the inhibitor suggesting double strand breaks in DNA, but not in IMR90. β-Actin loading control is shown below for each cell line. **B.** TUNEL staining (green) was strongly positive in the Capan-1 (2 μM PHA-767491) and PANC-1 (10 μM PHA-767491) cells but absent in the IMR90 treated cells (2 μM PHA-767491). DAPI staining (blue) is shown as a control. **C.** The proportion of cells in sub-G1 phase after 96 hours is shown on a bar chart for each cell line. Capan-1 shows high levels of sub-G1 accumulation at all dosages but in the PANC-1 line, there appeared to be a clear dose-dependent increase in the percentage of cells accumulating in the sub G1 peak (2 μM-15%, 5 μM- 22%, 10 μM- 49%) versus the DMSO and untreated controls.

Reduced levels of Mcm2 serine 53 phosphorylation was observed at all PHA-767491 drug concentrations in both pancreatic cancer cell lines and IMR-90 fibroblasts 96 hours following treatment with the inhibitor. Mcm2 protein phosphorylation became undetectable by western blot analysis at the 10uM concentration in PANC-1, and at the 2 μM concentration in Capan-1 and IMR-90; these concentrations were therefore used for further experiments. In Figure 6A, western blots are shown for each cell line. In the Capan-1 and PANC-1 cells, western blotting showed similar results to the *CDC7* siRNA-treated cells with increased expression of cleaved PARP-1 and Caspase-3 (89 KDa and 17 KDa fragments respectively), as well as induction of γH2A.X expression, a marker of advanced DNA fragmentation. IMR-90 cells, as in previous experiments with *CDC7* siRNA, showed no evidence of activation of the apoptotic machinery.

TUNEL staining was again strongly positive in the Capan-1 and PANC-1 treated cells, but absent in the IMR-90 and DMSO treated control cells, consistent with the findings from flow cytometry (Figure 6B). Phase contrast microscopy showed widespread cell death of both PANC-1 and Capan-1 cells treated with PHA-767491. In contrast, IMR-90 cells treated with either PHA-767491 or *CDC7* siRNA exhibited normal cell morphology but a much smaller population of cells was observed compared with controls consistent with a viable G1 cell cycle arrest as observed in previous studies (Figure 4) [19, 21, 23].

In Figure 6C, the proportion of cells in sub-G1 phase is shown on a bar chart for each cell line, showing that by 96 hours there Cdc7 kinase inhibition was associated with marked cell death in both the Capan-1 and PANC-1 cell lines at 96 hours, indicated by the accumulation of cells with a sub-G1 DNA content, around 70% and 49% for the two pancreatic cell lines respectively at the 10uM concentration. Interestingly a dose dependent increase in cancer cell killing was observed for the PANC-1 cell line corresponding to the dose dependent decrease observed in Mcm2 serine53 phosphorylation. In contrast, untransformed IMR-90 cells treated with PHA-767491 showed minimal cell death with the majority of cells arrested in the G1 peak, mirroring the G1 cell cycle arrest observed with Cdc7 siRNA (Figure 5) and in keeping with previous studies [19, 21, 23].

## DISCUSSION

The target validation and proof of concept studies conducted here have identified Cdc7 kinase and the associated DNA origin activation checkpoint as a promising new therapeutic point in pancreatic cancer. We have shown via immunohistochemistry that pancreatic cancer specimens contain significantly higher expression levels of the target protein Cdc7 than resected tissue from patients with benign pancreatic disease. Furthermore, we have also shown that the expression of Cdc7 strongly correlates with 3 other markers for cell cycle progression (Mcm2, geminin and phosphohistone H3) in pancreatic cancer. Following *CDC7* knockdown with siRNA, pancreatic adenocarcinoma cells undergo pronounced apoptotic cell death. These findings are in keeping with the cancer-cell-specific killing observed following Cdc7 targeting in other malignancies including leukemia and breast, ovarian and colorectal cancers [19–22, 25]. The cell lines tested, PANC-1 and Capan-1, harbour mutations in the tumour suppressors *TP53*, *CDKN2A, CDKN2B* and *CDKN1B* encoding for p53, p14^ARF^ and p16^INK4A^, p15^INK4B^ and p27^Kip1^, key constituent proteins encompassing all three arms of the origin activation checkpoint and representing mutations commonly found in pancreatic adenocarcinoma (Figure 1). In response to loss of function of any of these effector proteins we have previously shown that there is abrogation of the DNA origin activation checkpoint [23]. This results in tumour cells circumventing the G1 arrest thereby progressing into an abortive S phase. In the presence of rate limiting levels of Cdc7 kinase, only a limited number of replication forks are established during S phase, culminating in fork stalling and/or collapse followed by apoptotic cell death [19–22]. Notably the potent cancer cell specific killing observed in pancreatic cancer following Cdc7 inhibition or depletion contrasts with the stable G1 arrest observed in untransformed normal human diploid fibroblasts (IMR-90) with an intact DNA origin activation checkpoint and mirrors the G1 cell cycle arrest observed in previous studies with primary untransformed cell lines [9, 21, 23]. Moreover, we have also observed a stable reversible G1 arrest in normal (HMEpC) and immortalised (MCF10A) human mammary (HMEpC) epithelial cells following Cdc7 depletion [19]. Taken together these data highlight the highly specific nature of the cancer-cell-specific killing following Cdc7 targeting and therefore make it a potentially powerful target in the treatment of advanced pancreatic cancer.

Interestingly, p53 inactivation not only abrogates the DNA origin activation checkpoint but also increases the Cdc7 protein expression levels of the target protein itself, thus potentially reinforcing therapeutic efficacy through a positive feedback loop [24]. Intriguingly we have now discovered the mechanism linking loss of p53 function to increased Cdc7 levels. We have shown that p53 is a negative regulator of Cdc7 and that this linkage is part of the generalized p53 cellular stress response. Following genotoxic insult it is well established that p53 levels increase resulting in induction of p21 which blocks DNA replication initiation through inhibition of CDK2 function. We have now shown that a parallel effector pathway blocks DNA replication initiation by lowering Cdc7 levels. We have discovered that p53 controls Cdc7 stability post-transcriptionally via miR-192/215 and post-translationally via Fbxw7β E3 ubiquitin ligase (Tudzarova et al, manuscript submitted). Thus loss of p53 function attenuates this negative regulation of Cdc7 levels and is thus in keeping with the observation that Cdc7 levels are raised in p53 mutant tumours [24].

In summary, we have shown that Cdc7 kinase is highly expressed in pancreatic adenocarcinoma and therefore a potentially attractive target in this tumour type. Furthermore, targeting of Cdc7 in pancreatic cancer cell line model systems with either *CDC7* siRNA or alternatively Cdc7 SMI inhibitors can induce potent cancer cell-specific killing. Targeting Cdc7 using either of these modalities therefore offers new exciting therapeutic opportunities for the treatment of this aggressive disease for which treatment options are presently limited.

## MATERIALS AND METHODS

### Immunohistochemistry

Between 1/1/2005 and 1/1/2010 all patients who had had a biopsy or surgical resection sample of pancreatic tissue at University College Hospital, London were identified using the CoPath histology database (Sunquest, Tucson AZ, USA). Patients were excluded if the sample was inadequate for immunohistochemistry or if clinical data were not available. Formalin fixed paraffin embedded (FFPE) tissue blocks of representative tumour or benign tissue were obtained, and consecutive serial tissue sections were cut at a thickness of 4 μm onto Superfrost Plus slides (Visions Biosystems, Newcastle Upon Tyne, UK), dewaxed in xylene and rehydrated through graded alcohol to water. The tissue sections were pressure-cooked in 0.1 M citrate buffer at pH 6.0 for 2 minutes and immunostained using the Bond Polymer Refine Detection kit and Bond-III automated system (Vision Biosystems).

Primary antibodies were applied at the following dilutions: Mcm2 (1:1000), geminin (1:150), H3p (1:3000) and Cdc7 (1:100). Mcm2 monoclonal antibody (clone 46) was obtained from BD Transduction Laboratories (Lexington, KY, USA), Geminin monoclonal antibody from Leica Microsystems (Newcastle Upon Tyne, UK), Histone H3 phosphorylated on Serine 10 (H3p) polyclonal antibody from Upstate (Lake Placid, NY, USA), Cdc7 monoclonal antibody from MBL International (Woburn, MA, USA).

The slides were dehydrated with graded alcohol and then washed thrice with xylene (100% concentration). Incubation without a primary antibody was used as a negative control and tonsil epithelial sections as positive controls. Slides were initially evaluated at low-power magnification (100x) to identify the regions of tumour with the highest intensity of staining. From these selected areas, 3-5 fields at 400x magnification were captured with a charged-coupled-device camera and analysis software (SIS, Munster, Germany). Images obtained were printed in colour for quantitative analysis. After blinding for knowledge of clinicopathological variables, both positive and negative cells were counted within each field and stromal and inflammatory cells excluded. A minimum of 500 cells were counted for each case. The Labelling Index (LI) was then calculated for each marker using the following formula: LI= number of positive cells/total number of cells x100. Local research ethics committee approval was obtained from the joint UCL/UCLH Committees on the Ethics of Human Research (REC reference 06/Q0512/106).

### Cell culture experiments

Human pancreatic adenocarcinoma cell lines PANC-1 (ATCC; CRL-146) and Capan-1 (Capan-1 (ATCC; HTB-79), as well as the normal IMR-90 fibroblast line (ATCC; CCL-186) were used. The PANC-1 and Capan-1 pancreatic adenocarcinoma cell lines were selected as they represent cell line model systems representative of the majority of pancreatic cancers harbouring point mutations in the *TP53* gene as well as inactivating mutations in the tumour suppressor genes *CDKN2B, CDKN2A* and *CDKN1B* [26, 34–38]. Cells were cultured in BD Falcon (Franklin Lakes, NJ, USA) T75 flasks or six-well plates in a 5% CO_2_ incubator at 37°C. The medium used for culture was DMEM+ GlutaMAX™ High Glucose (Gibco, Invitrogen, Grand Island, NY, USA) and fetal bovine serum (Gibco) was added at a concentration of 10% (PANC-1 and IMR-90) or 20% (CAPAN-1). Each treatment experiment was carried out in triplicate.

### RNA interference

For *CDC7* siRNA treatment, a custom double-stranded siRNA with sequence: sense strand 5′-GCUCAGCAGGAAAGGUGUUUU-3′ and antisense strand 5′-AACACCUUUCCUGCUGAGCUU-3′ (Dharmacon, Thermo Scientific, Lafayette, CO, USA) was used, with non-targeting siRNA (Ambion, Invitrogen, Grand Island, NY, USA) as a negative control. To control for a non-target effect, an alternative *CDC7* siRNA was also used with the sequence: sense strand 5′-GCTCAGCAGGAAAGGTGTTTT-3′ and antisense strand 5′-AACACCTTTCCTGCTGAGCTT-3′ (Dharmacon, Thermo Scientific, Lafayette, CO, USA). For the PANC-1 and IMR-90 lines, siRNA was diluted to 10, 50 and 100 nM concentrations in Opti-MEM (Gibco), and complexed with lipofectamine RNAiMAX (Invitrogen, Grand Island, NY, USA) according to the manufacturer's instructions. Due to poor efficacy of standard transfection reagents in the Capan-1 cell line, transfection was carried out via electroporation using the Amaxa Cell Line Nucleofector Kit V (Lonza Cologne AG, Cologne, Germany) with 50 and 100 nM siRNA, according to the manufacturer's instructions [39].

### Cdc7 inhibitor treatment

The Cdc7/Cdk9 inhibitor PHA-767491 was supplied as a powder which was resolved in the solvent dimethyl sulfoxide (DMSO). In these experiments, PHA-767491 was added to the medium at concentrations of 2, 5, or 10 μM and DMSO alone was used as a control. Cells were then reincubated at 37°C in a CO_2_-controlled incubator and harvested at the defined time points as indicated above.

### RNA isolation and quantitative real-time (qRT)-PCR

Following collection of the cells, total RNA was extracted using the Purelink RNA mini kit (Ambion, Invitrogen, Grand Island, NY, USA) according to the manufacturer's instructions. The exon-flanking primer set for Cdc7 (forward, 5′-AACTTGCAGGTGGTAAAAAG-3′; and reverse, 5′-TGAAAGTGCCTTCTCCAAT-3′) was generated using PrimerQuest (IDT, Coralville, IA, USA) and supplied by Eurofin Genomics (Ebersberg, Germany). Using the Superscript III Platinum SYBR Green One Step qRT-PCR kit (Invitrogen, Grand Island, NY, USA), RNA was reverse transcribed and amplifyed in one step. Fluorescent PCR products were detected using the Mastercycler ep Realplex 4 (Eppendorf, Hamburg, Germany). The qRT-PCR protocol was as follows: a single cDNA synthesis step case was performed at 50°C for 3 minutes; followed by a single denaturation step at 95°C for 10 minutes; then 45 cycles of denaturation at 95°C for 15 seconds, annealing at 47°C for 20 seconds and extension at 60°C for 20 seconds. The Eppendorf Realplex Detection System Software (Eppendorf, Hamburg, Germany) was used to determine Cycle threshold (Ct) values. GAPDH measurements were used to normalize the data and the relative expression of *CDC7* RNA in treated samples to untreated samples was determined.

### Immunoblotting

For western blot analysis, cells were harvested and lysed in modified RIPA buffer (50 mM Tris–HCl, 300 mM NaCl, 1% NP40, 0.5% sodium deoxycholate, 0.1% SDS, 1 mM EDTA and cOmplete Mini EDTA-free protease inhibitor cocktail by Roche Life Science) for 45 min on ice and then sonicated for 10 seconds as previously described [23]. Protein concentration was determined using the Bio-Rad DC protein assay (Bio-Rad, Hercules, CA, USA) according to the manufacturer's instructions. Protein concentrations were analysed in comparison to a pre-prepared standard curve using a MDS Analytical Technologies SpectraMax MZ plate reader (Molecular Devices, Sunnyvale, CA, USA). Following denaturing, samples containing 40 μg total protein were loaded into each well with gel loading buffer onto Novex 4-20% Tris-Glycine 1.5 mm, 15 well gels (Invitrogen, Grand Island, NY, USA). Blocking, antibody incubations and washing steps were performed as described [40]. Protein was transferred onto Amersham Biosciences Hybond-C Extra membranes (GE Healthcare, Buckinghamshire, UK) by semi-dry electroblotting at 15V for 60 minutes, according to the manufacturer's instructions and developed using ECL Chemiluminescent Substrate Reagent Kit (Invitrogen, Grand Island, NY, USA).

### Immunoblotting antibodies

Mcm2 phospho Serine (Ser) 53 polyclonal antibody was obtained from Bethyl Laboratories (Montgomery, TX, USA) and used at a concentration of 1:500; Cdc7 monoclonal antibody (clone DCS-342) from MBL International (Woburn, MA, USA) and used at a concentration of 1:1000; caspase 3 (CPP32 4-1-18) monoclonal antibody from Novus Biologicals (Littleton, CO, USA) and used at a concentration of 1:1000; cleaved caspase 3 (Asp175) polyclonal antibody from Cell Signaling Technology Inc. (Danvers, MA, USA) and used at a concentration of 1:1000; PARP-1/2 (H-250) polyclonal antibody from Santacruz Biotechnology (Santa Cruz, CA, USA) and used at a concentration of 1:1000; Phospho-Histone H2A.X (Ser139) Antibody from Cell Signaling Technology Inc. (Danvers, MA, USA) and used at a concentration of 1:1000; β-actin from Sigma Aldrich (St. Louis, MO, USA) and used at a concentration of 1:5000.

### Cell sorting (FACS analysis)

Following collection, the cells were pelleted and fixed in 80% methanol at −20°C for at least 1 hour. Prior to FACS, cells were incubated with FACS solution containing 50 ug/ml propidium iodide (PI) and 50 ug/ml RNase A at 37°C for 1 hour and then sorted on a Beckman Coulter CyAN ADP flow cytometry system (Beckman Coulter Inc., Orange County, CA, USA). The forward scatter signal was used to trigger the detection of cells and the PI signal fluorescence was linearly quantified to rationalise DNA after excitation at 488 nm. PI fluorescence was detected on the FL3/PE-Texas Red detector (613/20 nm band pass filter). Gating was carried out as described to discount any surviving non-diploid cells.

### BrdU cell proliferation assay

Cells were cultured on coverslips in 6-well plates and treated as described above. Treated or control cells were pulsed with 100 μM 5-bromo-2′-deoxyuridine (BrdU) (Sigma Aldrich, St. Louis, MO, USA) for 1 hour prior to harvesting. Coverslips were then washed in 1 ml PBS and fixed with 4% paraformaldehyde. The cells were permeabilised in 0.2% Triton X for 5 minutes and 2N HCl was added for 1 hour to denature the cellular DNA. Coverslips were incubated in the dark at 37°C with 20 μg/ml fluorescein conjugated anti-BrdU antibody (Millipore, Temecula, CA, USA), 50 ng/ml PI solution and 50 ng/ml RNaseA. The coverslips were mounted onto slides and the cells examined with a Leica TCS SP confocal microscope.

### TUNEL assay

The terminal deoxynucleotidyl dUTP nick end labelling (TUNEL) assay was used for determining apoptosis in the cultured cells. Cells were grown on coverslips in 6-well plates and treated as described above. Coverslips were removed from the cell media at the designated time points fixed in 1% paraformaldehyde. Cells were permeabilised by submersion in precooled ethanol and acetic acid in a 2:1 mix for 5 minutes at −20°C. The ApopTag Fluorescein Direct *In Situ* Apoptosis Detection Kit (Chemicon, Millipore, Temecula, CA, USA) was then used according to the manufacturer's instructions. The coverslips were counterstained using PI solution and mounted onto glass slides for analysis with the Leica TCS SP confocal microscope.

### Annexin V assay

The Annexin V assay was carried out using the Millipore Muse Cell Analyser (Millipore, Temecula, CA, USA), according to the manufacturer's instructions. Briefly, 100 μg/ml cells were pelleted, before adding 100 μl of Annexin reagent and incubated in the dark for 20 minutes at room temperature. Annexin V labelled cell counts were then carried out in an automated fashion by the Muse Cell Analyser.

## SUPPLEMENTARY FIGURES AND TABLE


