# Supplementary material for: Cdc7 is a potent anti-cancer target in pancreatic cancer due to abrogation of the DNA origin activation checkpoint
Source: Oncotarget. 2016 Feb 23;7(14):18495–507. doi: 10.18632/oncotarget.7611 (PMC4951304; doi:10.18632/oncotarget.7611)
Supplement: Supplementary file 1 [file oncotarget-07-18495-s001.pdf]

SUPPLEMENTARY FIGURES AND TABLE

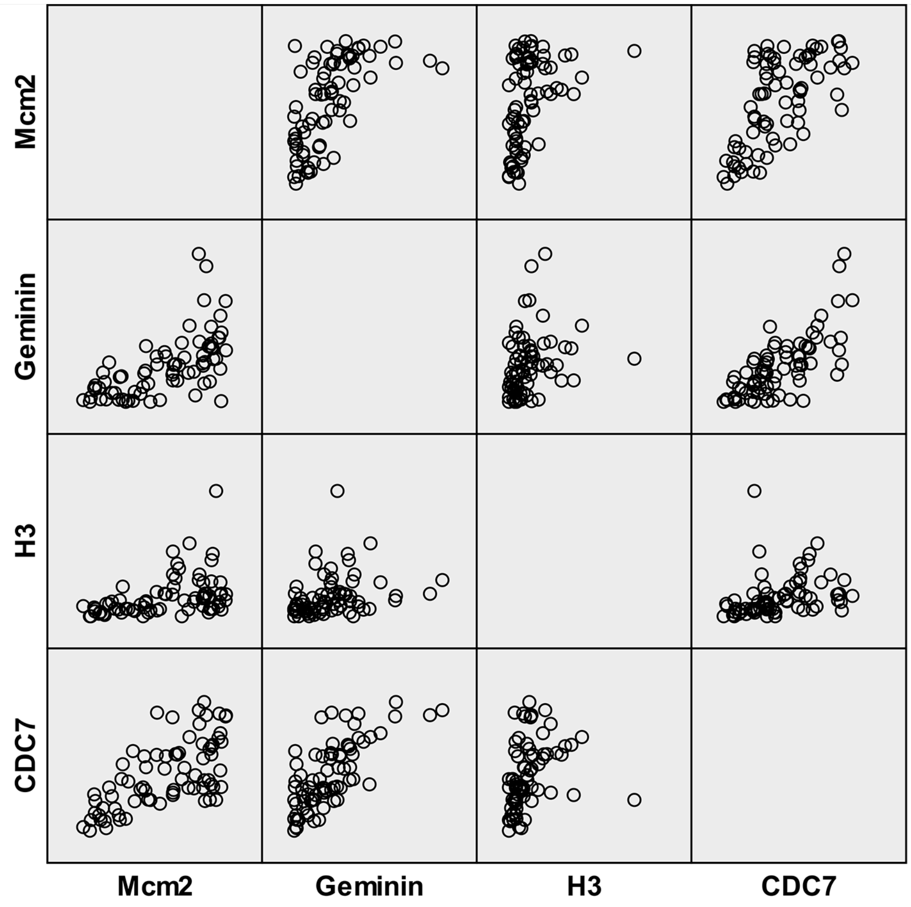

Correlations

			Mcm2	Geminin	H3	CDC7
Spearman's rho	Mcm2	Correlation Coefficient	1.000	.682	.505	.653
		Sig. (2-tailed)	.	.000	.000	.000
		N	73	73	73	73
	Geminin	Correlation Coefficient	.682	1.000	.429	.709
		Sig. (2-tailed)	.000	.	.000	.000
		N	73	73	73	73
	H3	Correlation Coefficient	.505	.429	1.000	.497
		Sig. (2-tailed)	.000	.000	.	.000
		N	73	73	73	73
	CDC7	Correlation Coefficient	.653	.709	.497	1.000
		Sig. (2-tailed)	.000	.000	.000	.
		N	73	73	73	73

Supplementary Figure S1: Spearman's Rank correlation coefficient was used to assess the association between Cdc7 and other markers for cell cycle progression. Cdc7, Mcm2, geminin and phosphohistone H3 were all found to be strongly associated with one another, with $P<0.0001$ for each correlation Analysis was using pairwise non-parametric correlation.

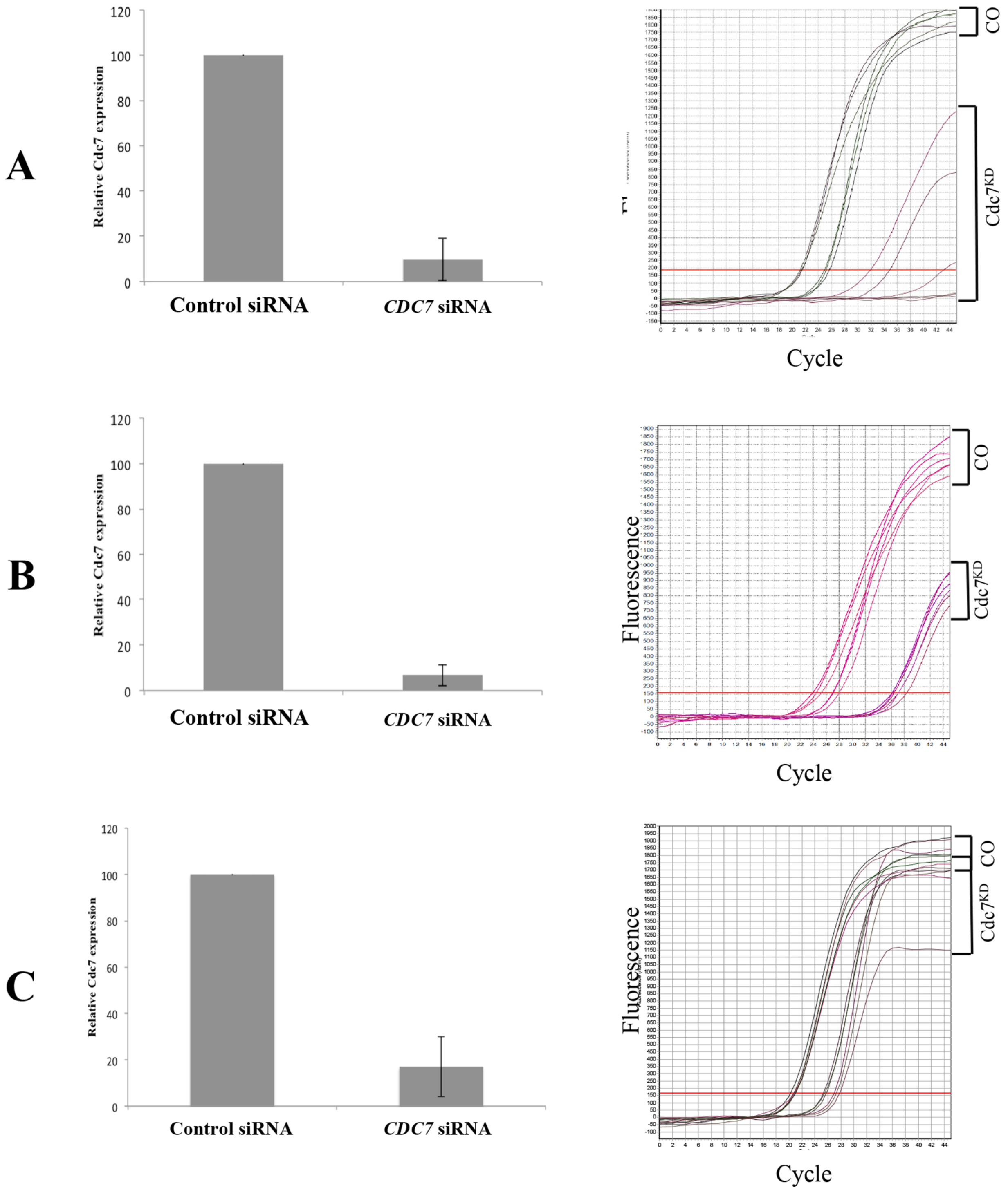

Supplementary Figure S2: Knockdown of *CDC7* mRNA in cell lines following transfection with custom siRNA. **A.** Relative expression and quantitative RT-PCR CT curves showing PANC-1 cells harvested at 48 hours post transfection with either *CDC7* siRNA or a control non-coding siRNA. **B.** Relative expression and quantitative RT-PCR CT curves showing Capan-1 cells harvested at 48 hours post transfection with either *CDC7* siRNA or a control non-coding siRNA. **C.** Relative expression and quantitative RT-PCR CT curves showing IMR-90 cells harvested at 48 hours post transfection with either *CDC7* siRNA or a control non-coding siRNA.

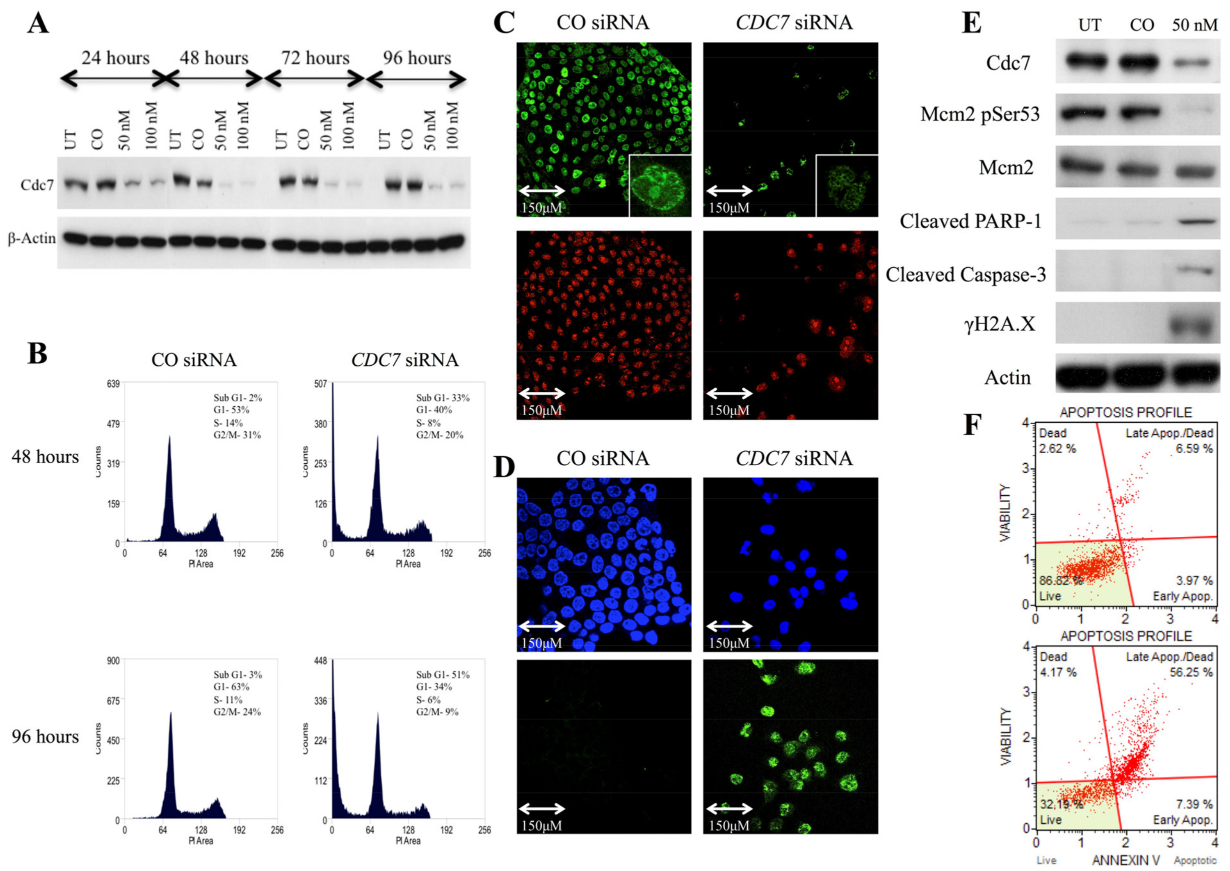

Supplementary Figure S3: Knockdown of *CDC7* mRNA in Capan-1 pancreatic adenocarcinoma cells following transfection with custom siRNA. A. Western blot showing Cdc7 protein expression levels following transfection with different concentrations of *CDC7* siRNA (50 and 100 nM) along with untreated (UT) and non-coding siRNA (CO). There was evidence of reduced protein expression at each concentration and at each time point. β-Actin loading control is shown below. **B.** Flow cytometry showing that at 48 hours, there was no enrichment of Capan-1 cells in the G1 population following treatment with 50 nM *CDC7* siRNA, and cells started to accumulate in a sub G1 peak. At 96 hours this effect was more pronounced with evident cell death, as represented by the sub G1 peak of 51%. **C.** BrdU staining (green) in cells treated with 50 nM *CDC7* siRNA and CO siRNA. A much smaller proportion of the *CDC7* siRNA cells stained positive, indicating reduced synthesis of new DNA. PI staining (red) is shown as a control. **D.** There was avid TUNEL staining (green) of 50 nM *CDC7* siRNA treated cells indicating apoptosis. DAPI staining (blue) is shown as a control. **E.** Western blot showing protein levels at 96 hours following Cdc7 depletion using 50 nM siRNA in the Capan-1 pancreatic adenocarcinoma cell line. There was reduced expression of Cdc7 protein and also loss of Cdc7 target phosphorylation of Mcm2 at Ser53. There was evidence of activation of the classical apoptotic pathway with cleavage of PARP-1 and Caspase-3. Phosphorylated γH2A.X was seen after Cdc7 depletion suggesting double strand DNA breaks. **F.** Annexin V staining confirmed apoptosis (early and late) in 11% of CO siRNA treated cells (upper graph), compared with 64% of the 50 nM *CDC7* siRNA treated cells (lower graph).

Supplementary Table S1: Association between Cdc7 and patient survival

Variable	Analysis	Hazard Ratio (95% CI)	P-value
Cdc7	Unadjusted	0.84 (0.74, 0.94)	**0.003**
	Covariate adjusted (†)	0.93 (0.81, 1.06)	0.26

In this analysis hazard ratios are given for a 10-unit increase in Cdc7 labelling index. The unadjusted result and those where the effects are adjusted for several potential confounding variables are summarised. All adjustments were performed using multivariate Cox regression analysis.

(†) Adjusted for sex, metastatic disease, ampullary vs. ductal adenocarcinoma, resection status and tumour differentiation
